# Case Report: Near-Fatal Intestinal Hemorrhage and Acute Acalculous Cholecystitis due to Vi-Negative and Fluoroquinolone-Insensitive *Salmonella enterica* Serovar Typhi Infection: A Rare Entity

**DOI:** 10.3389/fmed.2021.666643

**Published:** 2021-08-10

**Authors:** Yuehua Gong, Jianlin Li, Dongnan Zhu, Songsong Wang, Yingchun Xu, Yan Li, Yanqing Wang, Yan Song, Wenjuan Liu, Yunlong Tian

**Affiliations:** ^1^Yantai Center for Disease Control and Prevention, Yantai, China; ^2^Department of Radiology, The Affiliated Yantai Yuhuangding Hospital of Qingdao University, Yantai, China

**Keywords:** *Salmonella*, intestinal hemorrhage, acute acalculous cholecystitis, Vi-negative, antimicrobial resistant

## Abstract

Typhoid fever is usually a mild clinical disease. Typhoid fever with massive intestinal hemorrhage is rare in the antibiotic era. Acute acalculous cholecystitis (AAC) is also rare in adults. Here, we describe the first adult case of typhoid fever with both complications due to Vi-negative and fluoroquinolone-insensitive *Salmonella enterica* serovar Typhi (*S*. Typhi) infection. We aim to alert physicians to this rare condition.

## Introduction

Typhoid fever is a systemic infection caused by *S*. Typhi that causes an estimated 10.9 million new infections and 116,800 deaths worldwide each year (1–3). Typhoid fever usually presents with nonspecific symptoms such as malaise, fatigue, fever, abdominal pain, headache, and diarrhea or occasional constipation (4, 5). Despite improvement in treatment, 27% of hospitalized patients with typhoid fever continue to develop complications (6). The well-known complications of typhoid fever are intestinal hemorrhage and perforation, but these complications are not common in the current antibiotic era. Typhoid-induced acalculous cholecystitis rarely occurs, especially in adults.

Vi capsular polysaccharide is a major virulence factor and it is also a distinguishing feature of *S*. Typhi. It can increase the infectivity (7) of *S*. Typhi and the severity (8) of disease; however, Vi-negative isolates have been known for several decades (9).

Antimicrobial-resistant *S*. Typhi has been a global threat with the wide spread of multidrug-resistant (MDR) strains, defined as resistant to the traditional first-line antibiotics (ampicillin, chloramphenicol, and co-trimoxazole) since the early 1990s, and the emergence of extensively drug-resistant (XDR) strains, with resistance to the traditional first-line antibiotic fluoroquinolones and the third-generation antibiotic cephalosporins in the last 5 years (10).

This case highlights the need to raise our attention to those rare complications and antimicrobial resistance of *S*. Typhi for effective treatment of typhoid fever.

### Case Presentation

A 21-year-old Mexican patient presented to Donghai Hospital with complaints of fever (39.3°C), anorexia, and watery diarrhea three to four times a day. About 16 days prior to the onset of these symptoms, he arrived in Yantai, China. His condition improved after 2 days of antipyretic and antidiarrheal treatment (clindamycin 900 mg, etimicin 100 mg, anisodamine 10 mg, dexamethasone 5 mg, and antondine 2 ml). Unfortunately, the patient developed abdominal pain (paroxysmal colic) and diarrhea on the third day after discharge, and the symptoms gradually worsened. Two days later, the patient went to Yeda Hospital. On admission, he appeared ill and jaundiced but was alert. Initial vital signs were temperature 37°C, blood pressure 120/65 mmHg, heart rate 100 beats/min, and respiratory rate 18/min. Abdominal physical examination revealed right upper quadrant tenderness without rebound tenderness, liver percussive pain, Murphy's sign, no kidney percussive pain, and no rash. Initial investigation (Table 1) showed acute infectious, hyperbilirubinemia, elevated transaminases, and thrombocytopenia. The hepatitis panel was negative. Rapid tests for *Vibrio cholerae* O1/O139 were also negative. Chest computerized tomography (CT) showed bilateral inferior lobar foci, right pleural hypertrophy, and adhesion. Abdominal CT indicated hepatosplenomegaly and cholecystitis. Magnetic resonance cholangiopancreatography (MRCP) showed gallbladder enlargement and wall thickening, cholecystitis not excluded, bilateral pleural effusion, and a small amount of ascites. Blood culture revealed the growth of Gram-negative rod. After admission, he received a combined therapy of levofloxacin and imipenem. Due to his serious condition, he was transferred emergently to Yuhuangding Hospital at 9 pm the next day after admission.

**Table 1:** Summary of routine blood tests and blood biochemical tests.

**Investigations**	**Test in Yeda hospital**	**Test in Yuhuangding hospital**	**Normal range**
^a^WBC(10^9^/L)	6.18	6.44	3.5–9.5
^b^NEUT(10^9^/L)	Nil	5.38	2.5–7.5
^c^NEUT(%)	81.3	83.5	40–75
^d^LYM(%)	Nil	21.3	20–40
^e^EOS(10^9^/L)	Nil	0	0.05–0.5
^f^HGB (g/L)	Nil	135	120–160
^g^CRP (mg/L)	188	153.7	<5
^h^ALT(IU/L)	328	375	5–39
^i^LDH(IU/L)	Nil	1033	15–220
^j^AST(IU/L)	685	543	11–40
^k^ALP(IU/L)	776	736	35–135
^l^GGT(IU/L)	276	Nil	7–40
^m^DBIL (μmol/L)	109.9	Nil	0.3–8.6
^n^GLDH(IU/L)	112	Nil	0–1.5
°ALB(g/L)	29	Nil	38–51
^p^CK(U/L)	Nil	36	26–196
^q^PT (s)	Nil	15.2	11–13
^r^INR	Nil	1.32	2.0–2.5
^s^D-D (mg/L)	Nil	15.2	0–0.2
^t^PCT(ng/ml)	Nil	1.21	<0.05
^u^SAA (mg/L)	Nil	308.7	<10
^v^K (mmo1/L)	3.4	Nil	3.5–5.3
^w^PLT(10^9^/L)	24	23	125–350
^x^LEU	1+	Nil	Negative
^y^BIL	2+	Nil	Negative
^z^URO	1+	Nil	Negative

a *White blood cell count;*

b *Neutrophil count;*

c *Neutrophilic granulocyte percentage;*

d *Percentage of lymphocytes;*

e *Eosinopenia;*

f *Hemoglobin;*

g *C-reactive protein;*

h *Alanine transaminase;*

i *Lactate dehydrogenase;*

j *Aspartate transaminase;*

k *Alkaline phosphatase;*

l *Gamma glutamate transferase;*

m *Direct bilirubin;*

n *Glutamate dehydrogenase; °Albumin;*

p *Creatinine kinase;*

q *Prothrombin time;*

r *International normalized ratio;*

s *D-dimer;*

t *Procalcitonin;*

u *Serum amyloid A;*

v *Serum kalium;*

w *Platelet;*

x *Urine leukocyte;*

y *Urobilirubin;*

Z *Urobilinogen*.

**Figure 1:**
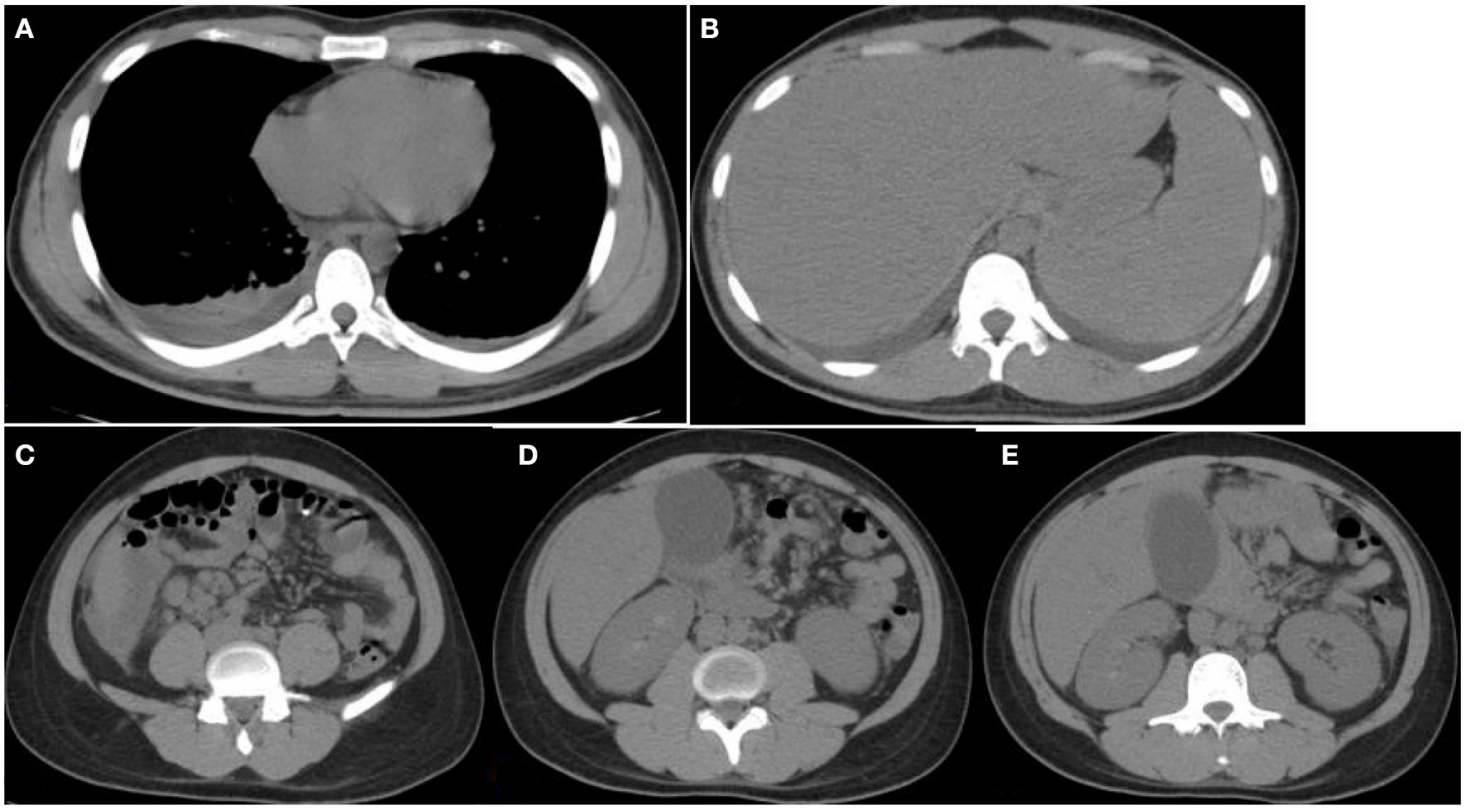
Chest and abdomen computerized tomography (CT) images. CT images obtained at Yuhuangding Hospital showing bilateral pleural effusion **(A)**, hepatosplenomegaly **(B)**, fluid collection around the right lower edge of the liver and multiple enlarged lymph nodes in the mesentery of the right lower edge of the liver, mesentery, and peritoneum of the middle abdomen **(C,D)**, gallbladder enlargement, and rough and thickened gallbladder wall **(E)**.

**Figure 2:**
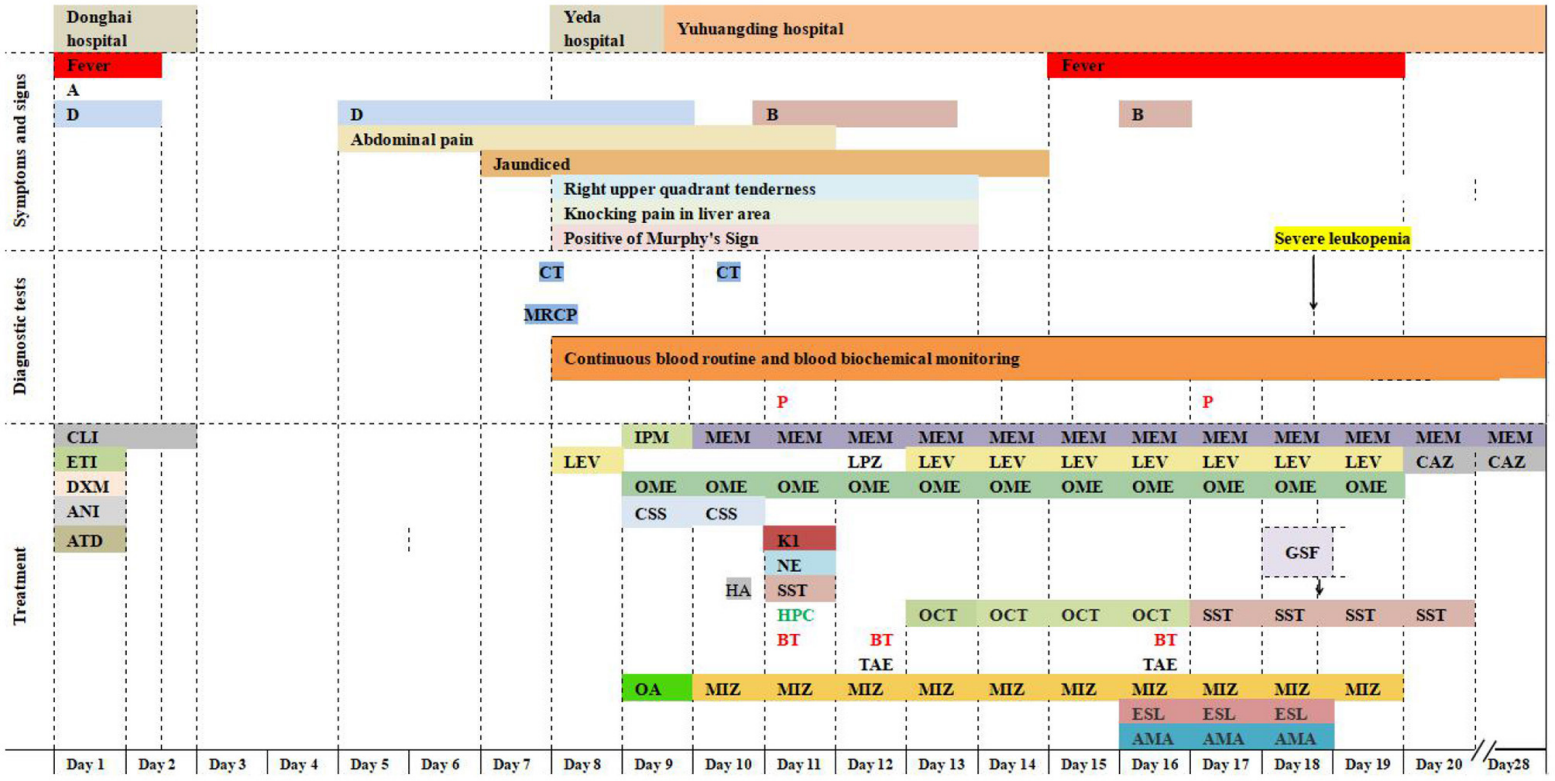
Timeline. Symptoms, diagnostic tests, and treatment. A, anorexia; B, intermittent bloody stools; D, intermittent diarrhea; CT, computed tomography; MRCP, magnetic resonance cholangiopancreatography; P, *S*. Typhi was isolated by blood culture; CLI, clindamycin 600 mg on day 1, 300 mg on day 2; ETI, etimicin 100 mg; DXM, dexamethasone 5 mg; ANI, anisodamine 10 mg; ATD, antondine 2 ml; LEV, levofloxacin 0.5 g/day; IPM, imipenem; MEM, meropenem 1 g/8 h; LPZ, lansoprazole 60 mg; OME, omeprazole 40 mg iv bid initially and then 40 mg iv qd; CSS, cefoperazone and sulbactam sodium 2 g/8 h; HA, hemocoagulase agkistrodon 2 IU iv; K1, vitamin K1 20 mg ivgtt; NE, norepinephrine 400 μg/h; OCT, octreotide 8 μg/h; SST, somatostatin 250 μg/h; HPC, human prothrombin complex 600 IU ivgtt; BT, blood transfusions 3.5 units on day 11, 12 units on day 12, and 4 units on day 16; OA, ornithine aspartate for injection 5 g; MIZ, magnesium isoglycyrrhizinate 150 mg/day during the first 4 days and then 100 mg/day; ESL, etamsylate 0.5 g/day; AMA, aminomethylbenzoic acid 0.1 g/day; GSF: recombinant human granulocyte stimulating factor 100 μg subcutaneous injection; TAE, transcatheter arterial embolization; CAZ, ceftazidime.

On arrival at Yuhuangding Hospital, his vital signs were temperature 35.8°C, blood pressure 114/59 mmHg, heart rate 89 beats/min, and respiratory rate 20/min. Blood tests (Table 1) were notable for elevated transaminases, prolonged prothrombin time (15.2 s, activity 53.9%), high international normalized ratio (1.32), elevated D-Dimer, elevated serum amyloid A and procalcitonin, thrombocytopenia, and eosinopenia. Chest CT showed bilateral pneumonia and bilateral pleural effusion. Abdominal CT showed fatty liver, hepatosplenomegaly, thickening of the horizontal duodenal wall, multiple enlarged lymph nodes in the mesentery, cholecystitis, and a small amount of ascites (Figure 1). The following test results were negative: the full panel for hepatitis; serologic tests for hantavirus, EB virus, and TORCH (*Toxoplasma gondii*, rubella, cytomegalovirus, herpes simplex virus, and other pathogenic microorganisms); nucleic acid assay for cytomegalovirus, EB virus, and the new Bunya virus; fecal bacterial isolation of *V. cholerae* O1/O139 and *Escherichia coli* O157:H7. The patient received intravenous meropenem 1.0 g per 8 h. On the second night after the transfer, the patient had bloody stools with clots three times within 3 h, about 50, 100, and 300 ml, respectively. Three urgent blood tests showed a continuous decrease in hemoglobin and platelet count from 126 g/L and 42 × 10^9^/L to 101 g/L and 26 × 10^9^/L, respectively. Blood pressure dropped to 96/45 mmHg. He was rendered norepinephrine (400 μg/h), somatostatin (250 μg/h), vitamin K1 (20 mg, ivgtt), and hemocoagulase agkistrodon (2 IU, iv). The patient had another bloody stool about 200 ml 8 h after the first bloody stool. The electrocardiograph (ECG) monitor showed blood pressure 115/54 mmHg, heart rate 107 beats/min, blood oxygen saturation 99%, and respiration 24 beats/min. Urgent blood test showed hemoglobin 83 g/L, platelet count 43 × 10^9^/L, prothrombin time 17.0 s, activity 46.0%, and fibrinogen 1.68 g/L. The patient received human prothrombin complex 600 IU and two infusions of O-type RH-positive RBCs (2.0 units and 1.5 units, respectively) without any transfusion side effects. On the third morning in Yuhuangding Hospital, Yeda Hospital reported the result of blood culture initiated at the time of admission, which was identified as *S*. Typhi. Transcatheter arterial embolization (TAE) refused by the patient initially was performed after 3 days of conservative treatment failed. Hemostasis was achieved and the patient continued to receive total parenteral nutrition and symptomatic support treatment (Figure 2). Unexpectedly, the patient had three more bloody stools (about 350 ml in total) with no obvious cause on the fourth day after TAE. Urgent blood test showed hemoglobin 66 g/L, neutrophil count 1.55 × 10^9^/L, K 3.22 mmol/L, Na 134.7 mmol/L, Ca 1.86 mmol/L, Mg 0.64 mmol/L, glucose 8.07 mmol/L, creatinine 48 μmol/L, urea 1.47 mmol/L, uric acid 157 μmol/L, prothrombin time 13.1 s, D-Dimer 12.6 mg/L, and procalcitonin 0.466 ng/ml. The patient was then transferred to the intensive care unit (ICU) and underwent TAE again. On the second day in the ICU, he had bloody diarrhea again. The hemoglobin level increased from 66 to 97 g/L after 4.0 units of RBC transfusion. On the third day in the ICU, the patient developed severe leukopenia (1.78 × 10^9^/L) and was treated with recombinant human granulocyte stimulating factor (GSF; 100 μg subcutaneous injection) promptly. After 5 days of intensive care in the ICU, his vital signs stabilized. The patient was discharged after another week of comprehensive treatment in the general ward. He returned to Mexico immediately after discharge and did not receive follow-up examinations as prescribed.

### Epidemic Management and Measures

The local center for disease control and prevention immediately conducted an epidemiological investigation after receiving the report of the outbreak from Yeda Hospital. The patient was quarantined. Fifteen close contacts received fecal bacterial isolation of *S*. Typhi (all tests were negative) and prophylactic medication. These close contacts were monitored dynamically for early detection of suspicious symptoms. The dormitory and workplace of the patient had been thoroughly disinfected.

### Referral of Bacterial Isolate to Yantai CDC

The *S*. Typhi isolate had been sent to Yantai CDC for further study. The phenotype, genotypic characteristics, and molecular typing of the isolate were studied, as described below.

### Phenotypic Characterization of Bacteria

The strain was identified as *Salmonella enterica* serovar Typhi and O9, 12: Hd serotype, using the API-20E automated microbial identification system and the White–Kauffmann–Le Minor Scheme serotyping techniques. Antimicrobial susceptibility test was performed with the AST Panel for Aerobic Gram Negative bacilli (Shanghai Xingbai Co.), referring to the interpretation of antimicrobial susceptibility of the Clinical and Laboratory Standards Institute (CLSI). The result showed that the strain was resistant to nalidixic acid, ampicillin, streptomycin, and polymyxin E and intermediate sensitive to levofloxacin, ciprofloxacin, ampicillin/sulbactam, cefazolin, and minocycline (Table 2).

**Table 2:** Resistotype of the *S*. Typhi isolate from the patient with typhoid fever.

**Antibiotic**	**Drug concentration(μg/ml)**	**MIC (μg/ml)**	Sensitivity^*^
Ampicillin	2–64	>64	R
Ampicillin/Sulbactam	2/1–64/32	16/8	I
Cefazolin	0.5–16	4	I
Cefotaxime	0.25–8	<0.25	S
Ceftazidime	0.5–16	<0.5	S
Cefoxitin	2–64	<2	#
Imipenem	0.25–8	<0.25	S
Amoxicillin/Clavulanic acid	2/1–64/32	8/4	S
Cefotaxime/Clavulanic acid	0.12/4–4/4	<0.12/4	A
Ceftazidime/Clavulanic acid	0.25/4–8/4	<0.25/4	B
Aztreonam	1–32	<1	S
Cefepime	0.25–16	<0.25	S
Meropenem	0.06–4	<0.06	S
Tetracycline	1–32	4	S
Minocycline	1–32	8	I
Kanamycin	8–64	<8	S
Streptomycin	4–32	>32	R
Amikacin	4–128	<4	S
Gentamycin	1–32	<1	S
Levofloxacin	0.125–8	0.5	I
Nalidixic acid	4–64	>64	R
Ciprofloxacin	0.03–32	0.25	I
Chloramphenicol	2–64	4	S
Trimethoprim-sulfamethoxazole	0.25/4.75–8/152	0.5/9.5	S
Polymyxin E	0.5–16	4	R
Polymyxin B	0.5–16	2	S
Azithromycin	2–64	4	S

* *S, susceptible; I, intermediate; R, resistant; #, For Salmonella spp., first- and second-generation cephalosporins and cephamycins may appear active in vitro but are not effective clinically and should not be reported as susceptible. The combination of A and B showed negative results of the extended-spectrum β-lactamase (ESBL) detection tests*.

### PCR for the viaB Virulence of the *S*. Typhi Isolate

The viaB operon, which encodes the genes responsible for Vi antigen expression (11), was detected by PCR referring to the research of Hirose et al. (12). The result showed that the isolate lacked the viaB operon.

### Pulsed-Field Gel Electrophoresis (PFGE) Analysis of Bacteria

PFGE analysis of *Xba*I- and *Bln*I-digested genomic DNA was performed to investigate the genetic relatedness of this isolate with five isolates from other four typhoid outbreaks in Yantai during the same period (within 2 months).

**Figure 3:**
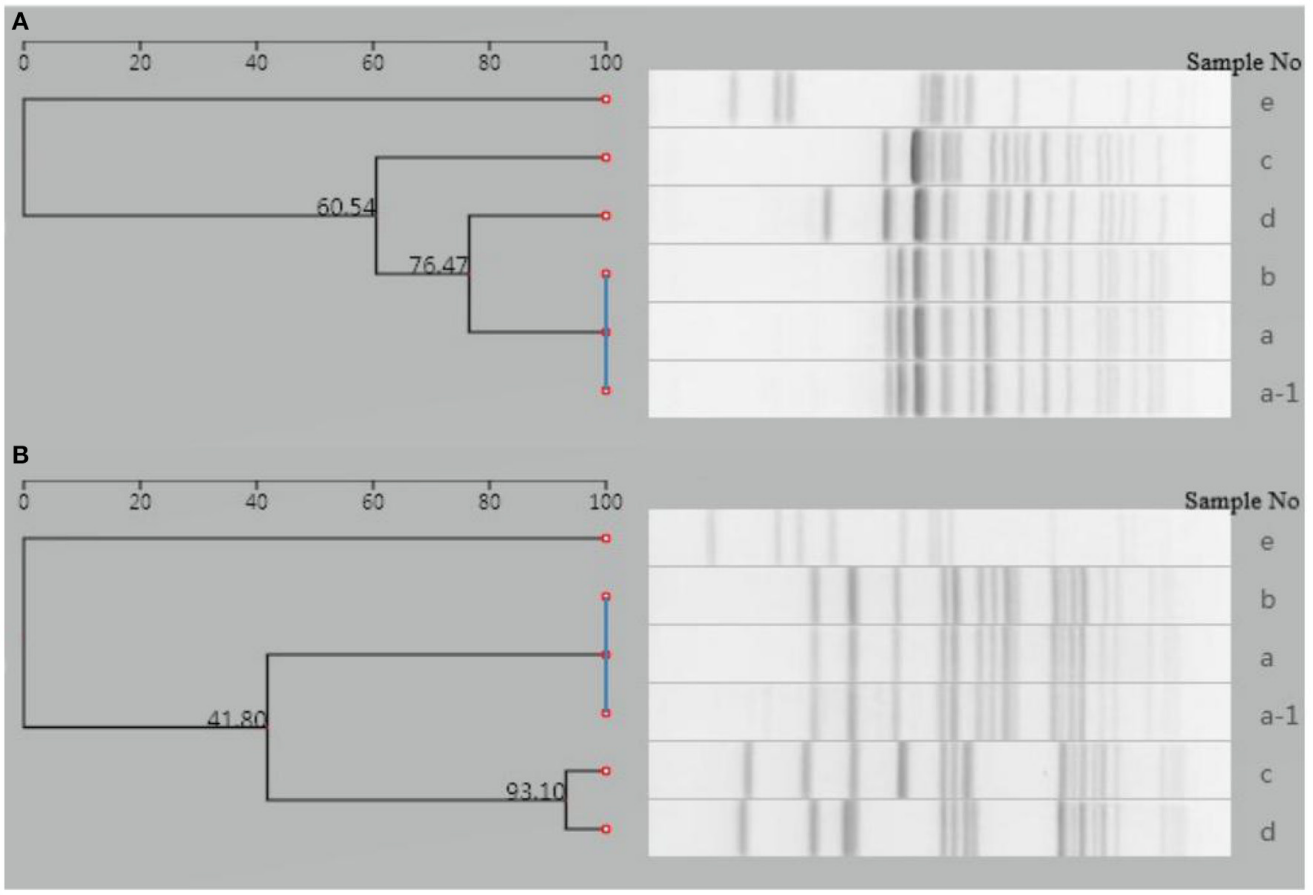
Dendrogram of PFGE patterns for six *S*. Typhi isolates detected in Yantai city, China. **(A)** Dendrogram of *Xba*I-PFGE patterns. **(B)** Dendrogram of *Bln*I-PFGE patterns. a-1, a–d: Isolates from another four typhoid outbreaks in the same period (within 2 months); e: isolate from the case presented in this study.

The analysis was performed using a Bio-Rad CHEF-DR III electrophoresis system (Bio-Rad Laboratories, Hercules, USA), following a PulseNet standardized protocol. Image data were imported into the national pathogenic bacteria identification network information system. The result showed that the isolate of the patient was distinguishable from the other five isolates (Figure 3), indicating that this isolate had no relatedness to the other four typhoid outbreaks. The PFGE fingerprint of this isolate was compared with all fingerprints of Shandong isolates (eight strains isolated from 2017 to 2018) in the system, and we found that this PFGE pattern had not been reported in Yantai or even in Shandong province.

## Discussion

We present a rare case of severe typhoid fever with massive lower intestinal bleeding, AAC, and ascites, due to Vi-negative and fluoroquinolone-insensitive *S*. Typhi infection. Considering an incubation period of 3 to 60 days (13) and the result of PFGE analysis, the infection was most likely acquired in Mexico or on the journey.

The diagnosis of typhoid fever may be difficult due to its reduced incidence and the appearance of atypical presentations. Hoshino et al. (14) observed a high prevalence of atypical presentations in typhoid fever. Traditionally, typhoid fever has been characterized by fever, relative bradycardia, rose spots, and splenomegaly, but these signs are frequently absent (15). Our patient did not develop the typical symptoms of rose spots and relative bradycardia. However, several clinical manifestations, such as an eosinophils absolute value of 0 and a normal white blood cell count despite high fever, were ignored. Typhoidal hepatitis occurs in the majority of patients (14). However, severe hepatic derangement similar to severe acute viral hepatitis is rare. Finally, a diagnosis of typhoid fever was established by the positive blood culture 10 days after onset. Isolation of the causative bacteria from blood remains the gold standard for the diagnosis of typhoid fever (16). Early diagnosis based on appropriate bacteriological inspection rather than typical symptoms should be stressed.

Vi capsular polysaccharide, which plays an important role in conferring resistance to the killing effect of serum, is a major virulence factor of *S*. Typhi (8). Despite the character of the Vi antigen as a distinguishing feature of *S*. Typhi, Vi-negative isolates have been reported in several countries (9, 17). Furthermore, there have been reports of typhoid outbreaks caused by Vi-negative *S*. Typhi (17), but the clinical symptoms were not described. Despite being less infective than Vi-positive *S*. Typhi, Vi-negative isolates can cause an indistinguishable typhoid fever from the one caused by Vi-positive isolates in volunteers (7). Vi antigen increases the infectivity (7) of the isolates and the severity of the disease (8). In this case, we found that Vi-negative *S*. Typhi-infected cases could also develop severe complications.

Our isolate was insensitive to the current first-line fluoroquinolones (intermediate sensitive to levofloxacin and ciprofloxacin, resistant to nalidixic acid), while it was sensitive to chloramphenicol and co-trimoxazole (traditional first-line antibiotics), cefotaxime and ceftazidime (third-generation cephalosporins), and azithromycin (macrolides). The study of Holt et al. (18) has shown that IncHI1 plasmids are responsible for the MDR phenotype of *S*. Typhi, and the use of antibiotics applies strong selective pressure on the maintenance of this plasmid. The reduction of selective pressure after discontinuation of traditional first-line antibiotics appears to induce the loss of the IncHI1 plasmids and the recovery of efficacy of these antibiotics. Zellweger et al. (19) emphasized the need to review the current treatment regimens for enteric fever and recommended a switch from fluoroquinolone monotherapy to macrolide or cephalosporin in combination with traditional first-line antibiotics, which had restored their efficacy. The WHO currently recommends fluoroquinolones, traditional first-line antibiotics, third-generation cephalosporines, and azithromycin for typhoid fever treatment (20).

In the pre-antibiotic era, intestinal hemorrhage was quite common especially in patients who had been ill for more than 2 weeks (13). With the advent of antibiotics, the incidence of this complication is on the decline. The terminal ileum is the common site of hemorrhage in typhoid fever since it has abundance of Peyer's patches in which infection induces hyperplasia, leading to possibly subsequent ulceration that can either perforate or erode a vessel causing the hemorrhage. In 2017, the state of Yucatan in southeastern Mexico witnessed a sudden and substantial jump in the number of typhoid cases, with 11% patients suffering from gastrointestinal bleeding, but the etiological characteristics of the isolates were not reported (21). Several literatures have described the relationship between duration of pre-hospital symptoms and risk of complications (6, 22). A short period of fever and thrombocytopenia on admission may be the early signs of complications (23, 24). Relative bradycardia may be a more common symptom in the early stages of uncomplicated typhoid fever (25). Severe typhoid fever with complications has been reported to be associated with age, sex, and intermediate susceptibility to ciprofloxacin, but it is controversial (24, 26). Intestinal hemorrhage is usually mild and can be treated conservatively, but massive or persistent bleeding probably requires further intervention such as TAE or surgery. TAE is now accepted to be performed in cases of acute or massive gastrointestinal bleeding or recurrent postoperative bleeding (27), especially in cases with greater comorbidities unfit for surgery (28). However, complications such as rebleeding and bowel ischemia should be considered (29). Research by Tarasconi et al. (28) shows that TAE is safe and effective, but TAE is no substitute for surgery. The decision should be made by the relevant endoscopists, interventional radiologists, and surgeons depending on an individual basis and the characteristics of the hemorrhagic lesions (28).

Acute acalculous cholecystitis is a life-threatening disorder that usually occurs after severe burns, sepsis, extensive trauma, cardiovascular disease, diabetes, heart attack, major surgery, malignancy, or prolonged parenteral nutrition (30), with a total mortality of 15% and a post-trauma mortality of 27% (31). Typhoidal AAC is rare, with a reported incidence of 1.7% (32). It was primarily reported in a pediatric population (33, 34). This rare complication may be linked to MDR or more virulent forms of *Salmonella* infection (35). A study by Alam Sher Malik (36) showed that children with splenomegaly, thrombocytopenia, or leukopenia were more likely to develop complications, including cholecystitis. The onset time of typhoidal AAC is variable. It can occur in the first week or the second week, or even during convalescence or relapse (33). Our patient presented with the typical Charcot's triad of fever, right sided abdominal pain, and jaundice. Fever, vomiting, right upper quadrant abdominal pain, and jaundice are important manifestations in the diagnosis of AAC (30). Ultrasonography and CT are very helpful in the diagnosis of AAC (31). The treatment of acalculous cholecystitis is in principle the same as that of calculous cholecystitis (31). However, acalculous cholecystitis tends to occur in patients with severe disease, and often has a poor prognosis due to the high risk of perforation and necrosis (30). Therefore, early cholecystectomy appears to be preferable to conservative treatment, but the choice of treatment should be made based on an individual basis (31).

## Limitations

Since the patient returned to Mexico soon after discharge, systematic follow-up could not be performed for accurate prognostic information.

## Conclusion

We report a case of typhoid fever complicated with massive intestinal bleeding and AAC due to Vi-negative *S*. Typhi infection. Hemostasis was achieved by TAE combined with comprehensive treatment. AAC is a rare complication of typhoid fever, which has been mainly reported in children, and it may also occur in adults, as in this case. This case highlights that Vi-negative *S*. Typhi infection can also lead to severe complications in typhoid fever cases.

## Data Availability Statement

The raw data supporting the conclusions of this article will be made available by the authors, without undue reservation.

## Ethics Statement

Written informed consent was obtained from the individual(s) for the publication of any potentially identifiable images or data included in this article.

## Author Contributions

YG, JL, YT, and DZ: design and literature search. YG and JL: first draft. All authors critical revision, editing, listed contributed to the article, and approved the submitted version.

## Conflict of Interest

The authors declare that the research was conducted in the absence of any commercial or financial relationships that could be construed as a potential conflict of interest.

## Publisher's Note

All claims expressed in this article are solely those of the authors and do not necessarily represent those of their affiliated organizations, or those of the publisher, the editors and the reviewers. Any product that may be evaluated in this article, or claim that may be made by its manufacturer, is not guaranteed or endorsed by the publisher.
